# SOCS3 deficiency drives the primed to naive pluripotency transition by sustaining STAT3 activation

**DOI:** 10.3389/fgene.2026.1857225

**Published:** 2026-07-20

**Authors:** Renhong Lu, Suoni Huang, Mingyang Du, Xiumiao Yang, Junyang Liu, Zheyi Lin, Jian Yang

**Affiliations:** 1 State Key Laboratory of Cardiology, Shanghai East Hospital, School of Medicine, Tongji University, Shanghai, China; 2 Jinzhou Medical University, Jinzhou, China

**Keywords:** EpiSCs, ESCs, pluripotent stem cells, reprogrammed naive ESCs, SOCS3

## Abstract

**Objectives:**

The transition between naive and primed pluripotency is governed by dynamic signaling networks and transcriptional circuits. While the janus kinase (JAK)/signal transducer and activator of transcription 3 (STAT3) pathway is the master driver of naive pluripotency, the intrinsic negative feedback mechanisms that restrict its activation in primed epiblast stem cells (EpiSCs) remain incompletely defined. This study aimed to characterize the functional role of Suppressor of Cytokine Signaling 3 (SOCS3) in the primed-to-naive pluripotency transition.

**Methods:**

CRISPR (Clustered Regularly Interspaced Short Palindromic Repeats)/Cas9 (CRISPR-associated protein 9) -mediated *Socs3* knockout (KO) was generated in mouse EpiSCs, followed by primed-to-naive reprogramming induction in 2i/LIF [LIF (leukemia inhibitory factor), PD0325901 and CHIR99021) culture system. Molecular and phenotypic changes were evaluated via quantative real time PCR (qRT-PCR), Western blot, flow cytometry and immunofluorescence. Multilineage differentiation as-says were performed to verify pluripotency, and the STAT3-specific inhibitor Stattic was used to confirm the pathway dependence of the reprogramming phenotype.

**Results:**

*Socs3* was highly expressed in naive embryonic stem cells (ESCs) but minimally detected in EpiSCs. *Socs3* deletion uncoupled the JAK/STAT3 negative feedback loop, causing sustained STAT3 Tyr705 phosphorylation that drove rapid and successful primed-to-naive conversion. The resulting reprogrammed naive ESCs (rnESCs) reactivated the core naive transcriptional network and acquired multilineage differentiation potential. *Socs3* deficiency also delayed exit from naive pluripotency, and Stattic treatment completely abrogated *Socs3* KO-mediated reprogramming.

**Conclusion:**

SOCS3 acts as a pivotal inducible barrier to the primed-to-naive pluripotency transition. Eliminating SOCS3-mediated negative regulation to sustain STAT3 activation is an effective strategy to overcome stem cell reprogramming barriers, providing a key target for the precise manipulation of pluripotent stem cell (PSC) fate.

## 1 Introduction

The fate transition of pluripotent stem cells (PSCs) is an intrinsic part of embryonic development (Mulas et al., 2021; Takahashi and Yamanaka, 2016). Pluripotency is not a static property but a continuum represented by at least two distinct metastable states: the naive state, represented by embryonic stem cells (ESCs) derived from the pre-implantation blastocyst, and the primed state, represented by epiblast stem cells (EpiSCs) derived from the post-implantation embryo (Du and Wu, 2024; Pera and Rossant, 2021). While naive cells possess unbiased developmental potential, primed cells exhibit restricted potency and are refractory to chimera formation (Kinoshita et al., 2021; Nichols and Smith, 2009). Recent advance has further delineated the pluripotency continuum during embryo development, highlighting the dynamic transitions between the naive state, the intermediate formative state, and the primed state (Furlan et al., 2023; Smith, 2017). Reverting primed EpiSCs back to the naive state is a fundamental challenge in stem cell biology, as this process requires overcoming robust signaling and epigenetic barriers that stabilize the primed cell fate (Liu et al., 2021; Yu et al., 2020; Lu et al., 2025).

The establishment and maintenance of the naive state rely on specific signaling cascades, most notably the activation of the STAT3 transcription factor *via* the LIF/gp130 receptor axis (He et al., 2018; Betto et al., 2021). We previously demonstrated that STAT3 activation is the limiting factor for reprogramming somatic cells and EpiSCs to the naive state (Yang et al., 2010). While forced overexpression of constitutively active STAT3 can drive this primed-to-naive transition, recent strategies increasingly favor the use of small molecules to precisely modulate cell fate and overcome epigenetic barriers, offering rapid and reversible control over signaling pathways (Jia et al., 2024; Bae et al., 2025; Umeyama et al., 2024). However, the endogenous molecular mechanisms that actively restrict STAT3 signaling and function in primed EpiSCs remain incompletely elucidated. The rapid progress in CRISPR/Cas9 technologies and precise gene editing has provided unprecedented opportunities to dissect these complex regulatory networks in stem cells (Lotfi et al., 2023; Zhang et al., 2023; He et al., 2025). Uncovering these intrinsic “braking” mechanisms is therefore crucial for precisely manipulating pluripotent cell fate transition.

Under physiological conditions, STAT3 activation triggers the expression of *Socs3*, which acts as a classical negative feedback inhibitor by binding to JAK and the gp130 cytokine receptor, thereby attenuating downstream JAK/STAT3 signaling (Blaszczak et al., 2024; Samad et al., 2025). This autoregulatory feedback loop maintains homeostatic balance of STAT3 activity in naive ESCs. However, in the context of primed-to-naive reprogramming, we hypothesized that this feedback mechanism might act as a kinetic ceiling, preventing STAT3 activity from reaching the threshold required to rewire the naive state signal regulation network. Recent studies have highlighted the complexity of factors regulating pluripotency exit and naive-state reprogramming, and a genome-wide CRISPR screen under stringent differentiation conditions has further identified SOCS3 as a core regulator of pluripotency exit alongside novel uncharacterized factors (Chen et al., 2026; Collier et al., 2022). Building on these findings, we postulate that *Socs3* deficiency may act as a bidirectional gatekeeper of pluripotency: promoting the entry of primed EpiSCs into naive state and preventing naive ESCs from exiting pluripotency to differentiate.

In this study, we systematically investigated the functional role of SOCS3 in the primed-to-naive pluripotency transition. We demonstrated that genetic ablation of *Socs3* in EpiSCs disrupted the negative feedback loop of JAK/STAT3, leading to sustained STAT3 phosphorylation, which was sufficient to drive successful reprogramming to naive ESCs. Our work integrates signal transduction logic with pluripotent cell fate plasticity, identifying SOCS3 as a key target for optimizing the regulation of PSC fate transition.

## 2 Materials and methods

### 2.1 Cell culture

*Oct4-GFP* EpiSCs, originally described in (Guo et al., 2009), were a kind gift from professor Guo Ge and professor Austin Smith (Living Systems Institute, University of Exeter, United Kingdom). EpiSCs were cultured in AF (20 ng/mL Activin A and 12.5 ng/mL FGF2) in N2B27 medium (Ying et al., 2003). ESCs or rnESCs were cultured on 0.2% (w/v) gelatin coated plates in 2i/LIF which comprised 10 ng/mL LIF, 1 μM PD0325901 and 3 μM CHIR99021 in N2B27. When cells reached 80%–90% confluency, the media was aspirated and the cells were dissociated with accutase for 3 min. Dissociated cells were spun down at 1,200 rpm for 3 min, resuspended in the corresponding fresh culture medium, and plated at a split ratio of 1:6 or at 1:8 split ratio. Cell lines were tested negative for *mycoplasma* detector kit (Vazyme, D101-01). All cell lines were cultured in a humidified incubator at 37 °C with 5% CO_2_.

### 2.2 Primed EpiSCs to naive ESCs reprogramming

For naive ESCs induction from primed EpiSCs: *Oct4-GFP* EpiSCs were separately plated into fibronectin coated 6-well plates and cultured in AF medium. After the cells reached 80% confluence, the medium was switched to 2i/LIF for 14 days to generate naive OCT4-GFP^+^ ESCs (rnESCs). The medium was refreshed every 2 days during the whole reprogramming process. OCT4-GFP^+^ naive ESC-like colonies were counted under the fluorescence microscope (Leica DMI4000) at days 13–14, and the reprogrammed colonies were further passaged at a split ratio of 1:6 to 1:8 in 2i/LIF on gelatin-coated plates for stability verification.

### 2.3 Exit from naive pluripotency

1 × 10^5^ rnESCs were plated into one well of 6-well gelatin-coated plates in 2i/LIF. After 24 h, medium was switched to N2B27 for 48 h. The cells were harvested for subsequent analysis.

### 2.4 Germ layer differentiation

5 × 10^5^ rnESCs were plated in a 6-cm petri-dish in M10 medium (KnockOut™ DMEM medium supplemented with 10% fetal bovine serum, 1× glutamine–penicillin–streptomycin and 0.1 mM β-mercaptoethanol) for 4 days, then dissociated with 0.05% Trypsin/EDTA and 1 × 10^5^ cells were plated on 6-well fibronectin coated plates in M10 for another 4 days. For neuronal differentiation, undifferentiated rnESCs were plated at 1 × 10^5^ in N2B27 on 0.2% gelatinized 6-well plates for 6 days. The medium was changed every other day.

### 2.5 Plasmids transfection and determination of gene editing types

*Socs3* knockout (KO) EpiSCs were established from *Oct4-GFP* EpiSCs, using CRISPR/Cas9 based method. Guide RNA (gRNA) targeting exon 2 of *Socs3* was cloned into pGL3-U6-sgRNA-PGK-puromycin vector. For reprogramming, *Socs3* gRNA (1 μg) was transfected with SpCas9 (3 μg) into EpiSCs using Lipofectamine 3000 (Invitrogen, L3000001) according to manufacturer’s instructions. SpCas9 and gRNA empty vector transfected EpiSCs served as control.

At 24 h post-transfection, cells were treated with 2 μg/mL puromycin for 72 h to eliminate untransfected cells. Surviving cells were seeded into 6-well plates via limiting dilution (800 cells per well) to isolate monoclonal cell lines. Individual monoclonal colonies were picked, expanded under routine EpiSCs culture conditions, and subjected to genomic DNA extraction for subsequent gene editing verification.

gRNA guided gene editing was verified by T7 Endonuclease I assay (Beyotime, D7080L), according to manufacturer’s instructions. In brief, 700 bp DNA fragment up- and down-stream of gRNA targeted site was amplified by genomic PCR (F: 5′-TTA​TCC​GCG​ACA​GCT​CGG​AC-3′; R: 5′-CCG​GCT​GGC​TCC​ACT​TGA​AA-3′). 200 ng PCR product was mixed with T7 endonuclease I reaction buffer, heated at 95 °C for 5 min, allowing the temperature down to 85 °C at 2 °C/sec, then down to 0.1 °C/sec. The hybridized PCR product was incubated with 1 μL T7 endonuclease I at 37 °C for 30 min. The digested product was separated by agarose gel electrophoresis with edited genes displaying bands with different sizes. *Socs3* gRNA sequence: 5′-ACT​TCG​GAC​GAG​GGT​TCC​GT-3’.

Next, the TA cloning method was used to analyze the specific gene editing types. Briefly, the PCR products were cloned into T-Vector (Takara, 3271) and transformed into competent *E. coli* cells. The positive colonies were picked for Sanger sequencing, and sequences were aligned to the wild-type *Socs3* to characterize the editing events.

### 2.6 Flow cytometry analysis

Cells were washed twice with PBS and dissociated with accutase at 37 °C, then washed with cold PBS, centrifuge at 1,200 rpm, 4 °C, 3 min, two times, followed by filtration through 40 μm cell strainer. BD FACS Arial II was used for analysis according to the manufacturer’s instructions. The data analysis was performed with Flowjo (version 10.4.0).

### 2.7 Quantitative real time PCR (qRT-PCR)

Total RNA was isolated using the RNA Clean and Concentrator™-5 (Zymo, R1013) following the manufacturer’s instructions. RNA concentration was measured on a spectrophotometer (Thermo Fisher). cDNA was synthesized with HiScript III 1st Strand cDNA Synthesis Kit (Vazyme, R312-01) and amplified with Taq Pro Universal SYBR qPCR Master Mix (Vazyme, Q712-02) on QuantStudio 6 Flex (Life Technologies). *Gapdh* was used as an internal control. All qRT-PCR primers used in this study are listed in Supplementary Table S1.

### 2.8 Immunofluorescence analysis

Cells were washed twice in PBS for 3 min each, and fixed in 4% paraformaldehyde (PFA) for 20 min, room temperature (RT). Cells were permeabilized and blocked in PBS with 5% (w/v) BSA, 0.1% (v/v) TritonX-100, and 3% (v/v) goat serum for 20 min, RT, and then washed three times in PBS containing 0.1% Triton X-100 (washing buffer). Cells were incubated with primary antibodies for 2 h, RT, and then washed three times. Cells were incubated with fluorescence conjugated secondary antibodies and 300 nM DAPI for 1 h, RT. After washing, cells were imaged with a fluorescence microscope. Antibodies used are listed in Supplementary Table S2.

### 2.9 Western blot

Cells were lysed with RIPA buffer supplemented with protease inhibitor (Roche) on ice. Cell lysates were cleared by centrifugation 1,200 rpm for 10 min at 4 °C, and the supernatant was recovered. Protein concentration was measured by the BCA method (Beyotime, P0011). 20 μg protein was fractioned on NuPAGE 10% Bis-Tris gel (Thermo Scientific, NP0301BOX) and electroblotted onto PVDF membrane. Blots were blocked with 5% no-fat milk, 0.1% Triton-X100 in TBS for 1 h, RT and incubated overnight with primary antibodies at 4 °C. The next day, after washing, the blots were incubated with fluorescence conjugated secondary antibodies for 1 h, RT and signals were detected with ChemiDoc Touch Imaging System (Bio-rad). The images were analyzed by ImageJ (version 1.53a). The antibodies used are listed in the Supplementary Table S2.

### 2.10 Statistical analysis

All data represented three replicates or where sample size (n) was ≥3 indicated in the figure legends. For comparisons between two groups, the two-tailed unpaired Student’s *t*-test was used for independent samples, and the two-tailed paired Student’s *t*-test was used for matched samples. Comparisons among values for groups greater than two were performed by one-way analysis of variance (ANOVA) followed by a Tukey’s *post hoc* test. Prism Version 9.0.1(128) (Graphpad) software was used for all statistical evaluations. P values less than 0.05 were considered statistically significant. Error bars, n and * represent standard deviations, the number of independent experiments, and significant difference (P < 0.05) from indicated control groups, respectively. Results were considered to be significant at *p* < 0.05 (*), *p* < 0.01 (**), *p* < 0.001 (***), *p* < 0.0001 (****); no significant (ns) at *p* > 0.05. At least three independent experiments were performed to confirm findings.

## 3 Results

### 3.1 SOCS3 is highly expressed in naive ESCs but minimally detected in primed EpiSCs

**FIGURE 1:**
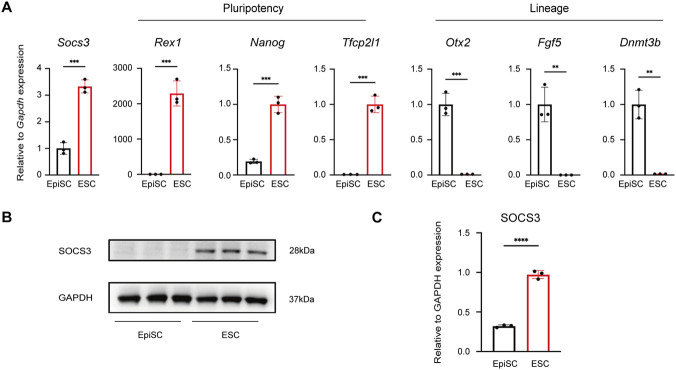
SOCS3 is highly expressed in naive ESCs but minimally detected in EpiSCs. **(A)** qRT-PCR analysis of *Socs3*, pluripotency and lineage marker genes in EpiSCs and ESCs. Gene expression was relative to *Gapdh*, normalized to EpiSCs or ESCs (n = 3, biological replicates). **(B)** Western blot analysis of SOCS3 protein expression in EpiSCs and ESCs. GAPDH served as loading control (n = 3, biological replicates). **(C)** Quantitative analysis of SOCS3 protein level in EpiSCs and ESCs. Pvalues (in this figure) by two-tailed unpaired Student’s t-test **(A) (C)** for comparisons between EpiSCs and ESCs. **p < 0.01, ***p < 0.001, ****p < 0.0001 indicate significant difference.

To investigate the potential role of SOCS3 in the dynamic regulation of pluripotent states, we first systematically characterized the basal expression profile of SOCS3, together with canonical naive and primed pluripotency markers, in stably cultured naive ESCs and EpiSCs. qRT-PCR analysis revealed a typical molecular signature of the two pluripotent states: naive ESCs exhibited robust expression of the core naive pluripotency markers *Rex1*, *Nanog* and *Tfcp2l1*, whereas EpiSCs highly expressed the primed state markers *Otx2*, *Fgf5* and *Dnmt3b*. Subsequently, we examined the expression pattern of *Socs3* in these 2 cell types and found that *Socs3* mRNA level was significantly higher in naive ESCs than in EpiSCs (Figure 1A). To confirm this at the protein level, we performed Western blot analysis, which further verified that SOCS3 was abundantly expressed in naive ESCs but only minimally detectable in EpiSCs (Figures 1B,C).

This expression pattern of SOCS3 is fully consistent with the canonical negative feedback regulatory mechanism of the JAK/STAT3 signaling pathway that we and other groups have previously revealed in PSCs (Huang et al., 2014; Boyle et al., 2009; Yang et al., 2010). JAK/STAT3 signaling directly induces *Socs3* transcription, and the encoded SOCS3 protein then acts as a negative feedback inhibitor to attenuate JAK/STAT3 activation. These results indicate that the low basal expression of SOCS3 in EpiSCs is a reflection of the inactive state of the JAK/STAT3 signaling in these cells, rather than an inherent inability of EpiSCs to express *Socs3*. Based on these observations, we hypothesized that *Socs3* may serve as a pivotal “inducible barrier” for the primed-to-naive pluripotency transition: upon the addition of LIF, a key reprogramming cue, EpiSCs would rapidly upregulate *Socs3* expression to clamp down on STAT3 signaling, thereby preventing the sufficient accumulation of Tyr705-phosphorylated STAT3 (pY705) that is essential for activating the core naive pluripotency transcriptional network.

### 3.2 *Socs3* depletion induces sustained STAT3 activation and elevates naive pluripotency in EpiSCs

**FIGURE 2:**
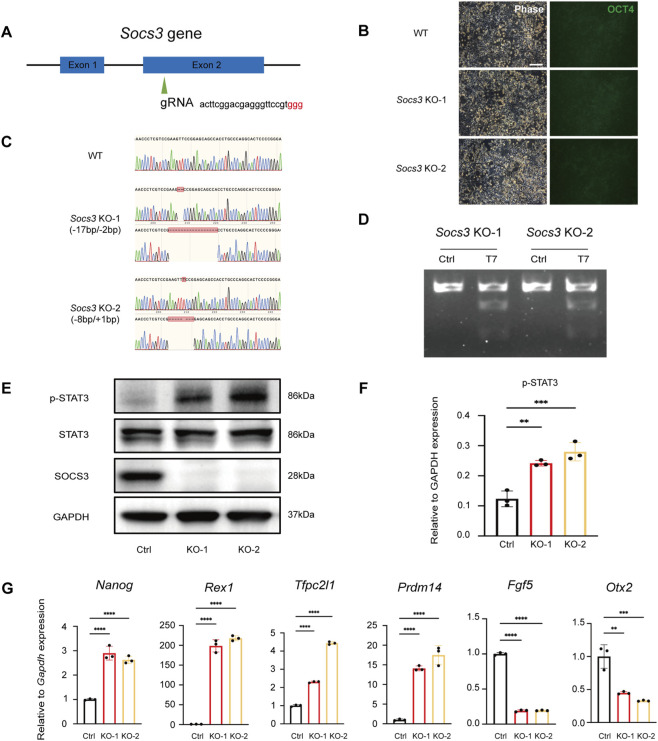
*Socs3* depletion induces sustained STAT3 activation and elevated naive pluripotency in EpiSCs **(A)** Schematic diagram of the mouse *Socs3* gene structure (Exon2) and the target sequence of gRNA. Protospacer-adjacent motif (PAM) is indicated in red. **(B)** Cell morphology and OCT4-GFP expression of WT and *Socs3* knockout (KO-1, KO-2) EpiSCs. Scale bar, 100 μm. **(C)** Agarose gel electrophoresis of T7 endonuclease I digestion product for validating *Socs3* KO gene editing in EpiSCs. Ctrl: EpiSCs without T7 endonuclease I treatment; T7: EpiSCs treated with T7 endonuclease I. **(D)** TA cloning and single-colony Sanger sequencing validating the gene editing types in *Socs3* KO-1 and KO-2 EpiSCs. **(E,F)** Western blot analysis of p-STAT3, STAT3, SOCS3 protein expression in WT, *Socs3* KO-1 and KO-2 EpiSCs. GAPDH served as loading control. **(G)** qRT-PCR analysis of pluripotency marker gene (*Nanog*, *Rex1*, *Tfcp2l1*, *Prdm14*) and lineage marker gene (*Fgf5*, *Otx2*) expression in WT, *Socs3* KO-1 and KO-2 EpiSCs. Relative to *Gapdh* expression, normalized to WT EpiSCs (n = 3, biological replicates). Pvalues (in this figure) were determined by one-way ANOVA **(F,G)** with Dunnett’s multiple comparison tests. **p < 0.01, ***p < 0.001, ****p < 0.0001 indicate significant difference.

To test our hypothesis that the SOCS3-STAT3 negative feedback loop constitutes the primary bottleneck for the primed-to-naive pluripotency transition, we generated *Socs3* KO *Oct4-GFP* EpiSCs *via* CRISPR/Cas9-mediated gene editing (Cong et al., 2013), with guide RNA (gRNA) specifically targeting the second exon of *Socs3* to ensure its functional ablation (Figure 2A). First, we characterized the basic cellular phenotype of *Socs3* KO EpiSCs under primed culture conditions and found that the cells maintained a typical flattened morphology of EpiSCs, which was indistinguishable from wild-type (WT) EpiSCs (Figure 2B), indicating that *Socs3* deletion does not alter the basal morphological characteristics of EpiSCs.

We then validated the efficiency and specificity of *Socs3* gene editing at the genomic level. The T7 endonuclease I assay was first performed for edited clones, and the results demonstrated clear cleavage of the *Socs3* gene at the targeted exon in both candidate cell lines (Figure 2C), indicating successful editing. Next, we performed TA cloning and single-colony Sanger sequencing of the targeted genomic region. Sequence analysis of multiple independent colonies revealed two distinct frameshift mutations per *Socs3* KO EpiSCs line, with no wild-type sequence detected. For KO-1 cell line: a 17-bp deletion and a 2-bp deletion were identified at the targetd locus; for KO-2 cell line: a 8-bp deletion and a 1-bp insertion were identified at the targetd locus, respectively (Figure 2D). Collectively, these results confirmed the successful establishment of two independent *Socs3* knockout EpiSC lines.

Next, we investigated the effect of *Socs3* deletion on the JAK/STAT3 signaling pathway. Western blot analysis and quantitative densitometry revealed that no detectable SOCS3 protein was present in either *Socs3* KO EpiSC line, confirming complete ablation of SOCS3 expression at the protein level, in stark contrast to WT EpiSCs with low p-STAT3, both *Socs3* KO EpiSC lines exhibited markedly elevated p-STAT3 protein, with no significant change in total STAT3 expression (Figures 2E,F). This result directly confirmed that deletion of *Socs3* uncouples the canonical negative feedback regulation of the JAK/STAT3 pathway, leading to sustained activation of STAT3 in EpiSCs.

To further explore the molecular consequences of sustained STAT3 activation in *Socs3* KO EpiSCs, we detected the expression of core naive pluripotency markers and primed associated markers via qRT-PCR (Figure 2G). The results showed that the transcription levels of *Nanog, Tfcp2l1, Rex1, Prdm14* were significantly upregulated in *Socs3* KO EpiSCs compared with WT EpiSCs, while *Fgf5* and *Otx2* expression was drastically downregulated. Collectively, these morphological, genomic and molecular data demonstrated that *Socs3* KO EpiSCs, while remaining in the primed culture medium, entered a metastable primed state with elevated naive pluripotency potential and sustained STAT3 activation—poised for rapid conversion to the naive pluripotent state upon appropriate induction.

### 3.3 *Socs3* deficiency drives EpiSCs to naive ESCs conversion

**FIGURE 3:**
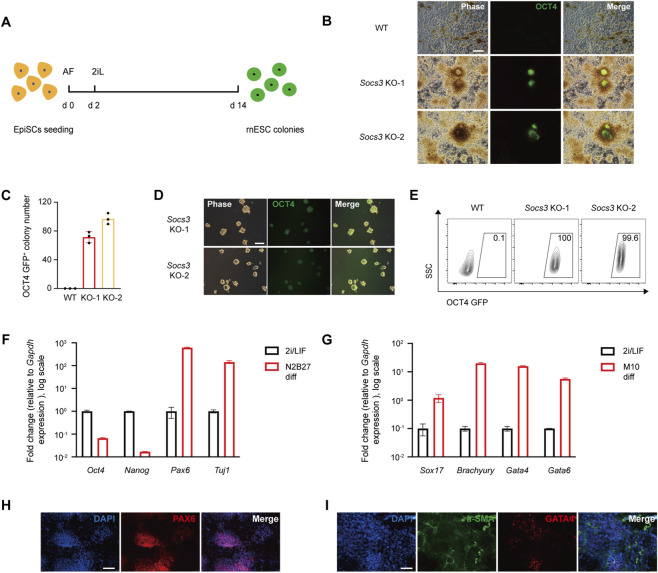
*Socs3* deficiency drives EpiSCs to naive ESCs conversion **(A)** Schematic diagram of the experimental workflow for reprogramming EpiSCs to rnESCs. **(B)** Primary *Oct4*-GFP^+^ colonies at day 14 in 2i/LIF. Scale bar, 100 μm. **(C)** Quantification of rnESC colony number generated from wild-type (WT), *Socs3* KO-1 and KO-2 EpiSCs after 14 days of reprogramming in 2i/LIF (n = 3, biological replicates). **(D)** Established *Socs3* KO *Oct4*-GFP^+^ rnESC line displayed naive ESC morphology. Scale bar, 100 μm. **(E)** Flow cytometry analysis of the *Oct*4-GFP^+^ cell proportion in *Socs3* KO-1 and KO-2 rnESCs. E14Tg2a ESCs (WT) served as negative control. **(F)** qRT-PCR analysis of pluripotency marker genes (*Oct4, Nanog*) and ectoderm lineage marker genes (*Pax6, Tuj1*) of *Socs3* KO rnESCs cultured in 2i/LIF and N2B27. Relative to *Gapdh* expression (n = 3, biological replicates). **(G)** qRT-PCR analysis of endoderm lineage marker genes (*Gata4, Gata6*) and mesoderm lineage marker genes (*Brachyury, Sox17*) of *Socs3* KO rnESCs cultured in M10 medium. Relative to *Gapdh* expression (n = 3, biological replicates). **(H)** Immunofluorescence staining depicting the localization of PAX6 (ectoderm marker, red) in differentiated *Socs3* KO cells. DAPI stains cell nuclei (blue). Scale bar, 100 μm. **(I)** Immunofluorescence staining depicting the localization of α-SMA (mesoderm marker, green) and GATA4 (endoderm marker, red) in differentiated *Socs3* KO rnESCs. DAPI stains cell nuclei (blue). Scale bar, 100 μm.

Given that *Socs3* deficiency EpiSCs exhibit sustained STAT3 activation (Wu et al., 2019) and a metastable primed state with elevated naive pluripotency potential, we next investigated whether *Socs3* KO alone is sufficient to drive the complete primed-to-naive transition in naive ESCs culture medium 2i/LIF (Yang et al., 2019; Stuart et al., 2019) (Figure 3A).

We cultured WT and two independent *Socs3* KO *Oct4-GFP* EpiSC lines in 2i/LIF and monitored cellular morphological changes and reprogramming dynamics over a 14-day time course (Figure 3B).

A striking phenotypic difference was observed between the two groups: WT EpiSCs failed to initiate reprogramming and instead underwent massive cell death and spontaneous differentiation. In sharp contrast, both *Socs3* KO lines underwent a rapid and progressive morphological transition. By day 14 of culture, the KO cells had formed highly refractive, dome-shaped colonies—the canonical morphological hallmark of naive ESCs, and expressed OCT4-GFP (Figure 3B). Quantitative analysis of reprogramming efficiency showed that approximately 100 OCT4-GFP^+^ colonies were generated from every 3 × 10^5^ plated *Socs3* KO EpiSCs (Figure 3C). Furthermore, these reprogrammed colonies could be efficiently expanded and stably propagated in 2i/LIF for multiple passages while maintaining the typical naive ESCs morphology (Figure 3D), demonstrating the stability of the reprogrammed naive state. We named these cells as reprogrammed naive ESCs (rnESCs). The flow cytometry results showed that the rnESCs exhibited complete expression of OCT4-GFP (Figure 3E).

This confirms that *Socs3* deletion effectively uncouples the signaling output from its negative regulator, mimicking the effect of hyperactive STAT3 constructs used in our previous studies (Yang et al., 2010), enabling EpiSCs to be reprogrammed to naive ESCs.

### 3.4 *Socs3* KO rnESCs possess multilineage differentiation potential

To exclude the possibility of aberrant cellular transformation and rigorously verify the functional pluripotency of the *Socs3* KO rnESCs, we assessed their ability to differentiate into three embryonic germ layers (ectoderm, mesoderm, and endoderm)—a defining feature of naive PSCs. In differentiation medium (10% fetal bovine serum) or N2B27 medium(Ying et al., 2003), the expression of pluripotency markers and germ layer-specific markers showed these cells exited naive state pluripotency and differentiated into mesoderm, endoderm, and ectoderm lineages (Figures 3F,G), with immunofluorescent staining confirming the expression of PAX6, α-smooth muscle actin (α-SMA) and GATA4 (Figures 3H,I). These functional assays unequivocally verified that the *Socs3* KO rnESCs possess authentic multilineage differentiation potential (Yang et al., 2019; Yu et al., 2021; Guo et al., 2024).

Collectively, these morphological, quantitative, molecular and functional data confirm that *Socs3* deletion effectively uncouples the JAK/STAT3 signaling pathway from its endogenous negative feedback regulation. This genetic modification mimics the reprogramming effect of hyperactive STAT3 overexpression, and is sufficient to enable EpiSCs to overcome the intrinsic barriers to naive pluripotency in 2i/LIF.

### 3.5 SOCS3 acts as a bidirectional gatekeeper of pluripotent state transition

**FIGURE 4:**
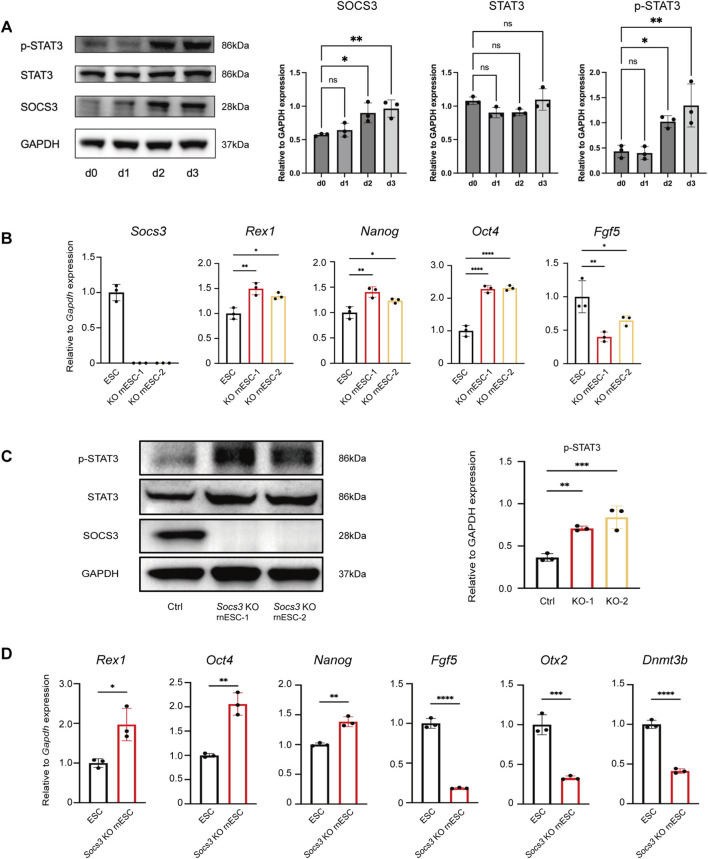
SOCS3 acts as a bidirectional gatekeeper of pluripotent state transition. **(A)** Western blot analysis of the dynamic nature of the SOCS3-STAT3 feedback loop. Quantitative analysis of SOCS3 and p-STAT3 protein level in EpiSCs and ESCs. GAPDH served as loading control (n = 3, biological replicates). **(B)** qRT-PCR analysis of *Socs3* and core pluripotency marker genes (*Rex1, Nanog, Oct4*) and differentiation marker gene *Fgf5* in WT ESCs and two independent *Socs3* KO rnESC clones. Relative to *Gapdh* expression, normalized to ESCs (n = 3, biological replicates). **(C)** Western blot analysis of p-STAT3, total STAT3 and SOCS3 protein expression in WT ESCs and two independent *Socs3* KO rnESC clones. GAPDH served as loading control (n = 3, biological replicates). **(D)** qRT-PCR analysis of pluripotency marker genes (*Rex1, Oct4, Nanog*) and differentiation marker genes (*Fgf5, Otx2, Dnmt3b*) in WT ESCs and *Socs3* KO rnESCs at differentiation day 2. Relative to *Gapdh* expression, normalized to ESCs (n = 3, biological replicates). Pvalues (in this figure) by two-tailed unpaired Student’s *t*-tests **(D)**, one-way ANOVA **(A–C)**, with Dunnett’s multiple comparison tests. ns indicates not significant, *p < 0.05, **p < 0.01, ***p < 0.001, ****p < 0.0001 indicate significant difference.

To directly validate this dynamic regulatory relationship, we performed a detailed time-course analysis of STAT3, p-STAT3 and SOCS3 expression during the first 3 days of primed-to-naive reprogramming: Western blot analysis revealed that phosphorylated STAT3 (p-STAT3) level began to increase from day 1 and continued to rise through day 3, while total STAT3 protein expression remained unchanged; SOCS3 protein level mirrorred this induction pattern, with robust expression detected from day 1 to day 3 (Figure 4A).

We next sought to determine whether SOCS3 deficiency also modulates the maintenance of and exit from naive pluripotency—an extension of our hypothesis that SOCS3 acts as a bidirectional gatekeeper of pluripotent cell fate. We first characterized the molecular and signaling features of *Socs3* KO rnESCs relative to WT naive ESCs in 2i/LIF. The qRT-PCR results showed that SOCS3 KO increased the pluripotency of the naive state (Figure 4B). Concomitantly, Western blot analysis and quantitative densitometry validated that the elevated naive pluripotency in *Socs3* KO rnESCs was accompanied by significantly increased p-STAT3, with no detectable change in total STAT3 protein expression (Figure 4C). These results indicate that SOCS3-mediated negative feedback regulation of STAT3 activation is not only critical for the primed-to-naive transition but also in the maintenance of naive pluripotent state.

The transition from naive pluripotency back to the primed state is the functional reverse of primed-to-naive reprogramming. Recent studies utilizing genome-scale screens have indicated that this process is tightly regulated (Gao et al., 2022; Zhou et al., 2024). To investigate whether SOCS3 governs the directionality of this transition. We induced pluripotency exit by culturing WT ESCs and *Socs3* KO rnESCs in N2B27. Under these conditions, WT ESCs rapidly downregulated pluripotency markers within 48 h. In striking contrast, *Socs3* KO rnESCs exhibited a “delayed exit” phenotype, maintaining significantly higher transcription level of naive markers. Concurrently, the induction of *Fgf5*, *Otx2* and *Dnmt3b* was significantly blunted (Figure 4D).

This finding reveals a dual role of *Socs3* deficiency in regulating pluripotent cell fate transition: it robustly promotes the primed-to-naive reprogramming by removing the inducible barrier to STAT3 activation, while markedly delays the exit from naive pluripotency and subsequent differentiation.

### 3.6 Sustained STAT3 activation is indispensable for reprogramming

**FIGURE 5:**
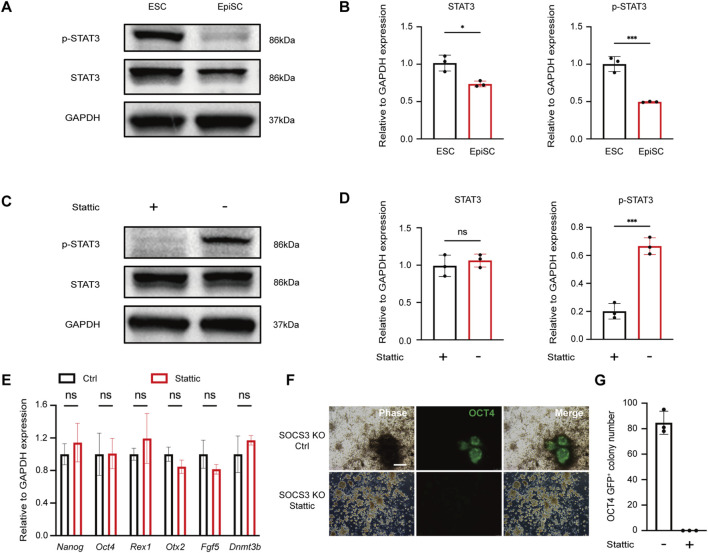
Sustained STAT3 activation is indispensable for reprogramming **(A,B)** Western blot analysis of phosphorylated STAT3 (p-STAT3) and total STAT3 protein expression in naive ESCs and EpiSCs. GAPDH served as loading control (n = 3, biological replicates). **(C,D)** Western blot analysis of p-STAT3 and STAT3 protein expression in *Socs3* KO EpiSCs with or without Stattic treatment (4 μm, 24 h). GAPDH served as loading control (n = 3, biological replicates). **(E)** qRT-PCR analysis of pluripotency marker genes (*Nanog, Oct4, Rex1*) and lineage marker genes (*Otx2, Fgf5, Dnmt3b*) expression in *Socs3* KO EpiSCs with or without Stattic treatment. Relative to *Gapdh* expression (n = 3, technical replicates). **(F)** Primary *Oct4*-GFP^+^ colonies from *Socs3* KO EpiSCs with or without Stattic treatment after 14 days of reprogramming in 2i/LIF. Scale bar, 100 μm. **(G)** Quantification of rnESC colony number generated from *Socs3* KO EpiSCs with or without Stattic treatment after 14 days of reprogramming in 2i/LIF (n = 3, biological replicates). Pvalues (in this figure) by two-tailed unpaired Student’s *t*-tests **(B,D,E)**. *p < 0.05, ***p < 0.001, indicated for significant differences. ns, not significant.

To definitively confirm that the primed-to-naive reprogramming driven by *Socs3* deficiency is strictly dependent on STAT3 activation, we used Stattic, a highly specific small molecule inhibitor of STAT3 that selectively abrogating pY705 (Guo et al., 2022; Zhong et al., 2025), and performed a series of experiments on EpiSCs reprogramming. We first validated the basal activation status of the JAK/STAT3 pathway in naive ESCs and EpiSCs, which showed that both total STAT3 and pY705 protein levels were significantly higher in naive ESCs than EpiSCs (Figures 5A,B), consistent with the canonical activation pattern of the JAK/STAT3 pathway in naive pluripotency.

We then treated *Socs3* KO EpiSCs with Stattic and assessed its effect on p-STAT3 level via Western blot. The results showed that Stattic treatment efficiently and completely abolished the sustained elevation of pY705 in *Socs3* KO EpiSCs, while having no discernible impact on total STAT3 protein expression (Figures 5C,D). To exclude the possibility that Stattic exerts non-specific effects on the basal expression of pluripotency markers, we further detected the transcription of core naive and primed markers in *Socs3* KO EpiSCs with or without Stattic treatment *via* qRT-PCR. No significant difference was observed in the expression of *Nanog*, *Oct4*, *Rex1*, *Otx2*, *Fgf5* and *Dnmt3b* between the two groups (Figure 5E), confirming that Stattic acts specifically on STAT3 phosphorylation without altering the basal gene expression of *Socs3* KO EpiSCs.

Finally, we investigated the impact of Stattic-mediated STAT3 inhibition on reprogramming by culturing Stattic-treated *Socs3* KO EpiSCs in 2i/LIF. Strikingly, the robust reprogramming phenotype driven by *Socs3* deletion was completely abolished upon Stattic treatment: the cells failed to undergo morphological transformation into dome-shaped naive colonies and lost the ability to activate OCT4-GFP expression (Figures 5F,G). These data provide compelling and direct evidence that sustained STAT3 activation is a decisive condition for *Socs3* deficiency mediated reprogramming. The reprogramming ability conferred by *Socs3* KO relies entirely on the activation of STAT3. Although the negative feedback barrier mediated by *Socs3* is lost, the reduction of STAT3 activation is sufficient to block the entire reprogramming process.

**FIGURE 6:**
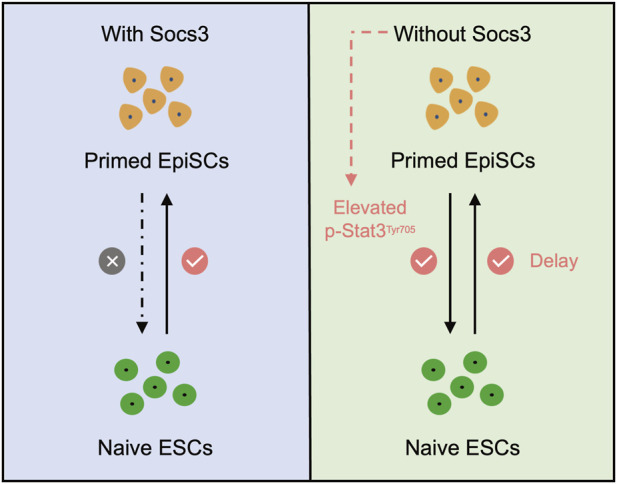
A hypothetical model for SOCS3 deficiency driving the primed to naive pluripotency transition by sustaining STAT3 activation. This schematic model illustrates the dual regulatory role of SOCS3 in controlling pluripotent state transition. In wild-type primed EpiSCs (left panel, with *Socs3*), the cells cannot be reprogrammed into naive ESCs, while ESCs can differentiate into EpiSCs. In contrast, genetic deletion of *Socs3* in EpiSCs (right panel, without *Socs3*) removes this signaling barrier, leading to sustained and elevated level of p-STAT3 Tyr705. The STAT3 activation drives the transcriptional rewiring required for the transition from the primed to the naive pluripotent state, resulting in the generation of naive ESCs. At the same time, it can also delay the exit from naive pluripotency.

This hypothetical model illustrates that SOCS3 acts as an inducible barrier to primed-to-naive pluripotency transition via negative regulation of STAT3. *Socs3* deficiency sustains STAT3 activation to drive efficient naive pluripotency reprogramming, while also bidirectionally gating pluripotent cell fate by delaying exit from naive state (Figure 6).

## 4 Discussion

Reverting EpiSCs to the naive state requires dismantling robust epigenetic and signaling barriers, a process typically achieved through multi-factor overexpression or complex chemical cocktails (Lu et al., 2025). Our study establishes SOCS3 as the critical inducible barrier governing the primed-to-naive pluripotency transition in mouse EpiSCs. CRISPR/Cas9-mediated *Socs3* deletion disrupts the JAK/STAT3 negative feedback loop, leading to sustained pY705 that drives efficient primed-to-naive conversion in 2i/LIF. The sustained activation of STAT3 upon uncoupling this feedback loop echoes recent findings that tight regulation of the IL-6/STAT3/SOCS3 axis is critical for dictating phenotypic and metabolic plasticity in various stem cell models (Blaszczak et al., 2024; Betto et al., 2021). Furthermore, recent multi-omics studies have highlighted that the transition between naive, formative, and primed states involves profound amino acid metabolic rewiring, RNA turnover, and dynamic changes in chromatin accessibility (Carbognin et al., 2023; Santini et al., 2024; McCreery et al., 2025; Park et al., 2023). Notably, *Socs3* deficiency exerts a STAT3-dependent dual role: it promotes primed-to-naive reprogramming and delays naive pluripotency exit, a phenotype completely abrogated by the p-STAT3 inhibitor Stattic. These results solidify the JAK/STAT3-SOCS3 axis as the core module controlling bidirectional pluripotent state transition (Bourillot et al., 2020).

Mechanistically, SOCS3’s differential expression—high in naive ESCs and inducible in primed EpiSCs, explains EpiSCs’ inherent resistance to LIF-mediated reprogramming (Stirparo et al., 2021). LIF stimulation rapidly upregulates *Socs3* in EpiSCs, which decreases p-STAT3 accumulation so that blocks pluripotency regulating network rewiring to the naive state (Stuart et al., 2014). This single-gene deletion strategy different from multi-small molecule reprogramming methods, aligning with CRISPR screening insights that targeting key negative regulators is highly efficient for pluripotency modulation (Gao et al., 2022).

Notably, this inducible feedback model also resolves a key mechanistic phenomenon: how *Socs3* deletion leads to robust STAT3 activation and naive marker induction in EpiSCs cultured in standard AF medium, which lacks exogenous LIF. This apparent discrepancy arises from a tightly regulated signaling circuit that maintains basal STAT3 activity at a precisely calibrated level in primed EpiSCs. We previously detected STAT3 Tyr705 phosphorylation in EpiSCs without exogenous LIF (Yang et al., 2010). This basal activity may be mediated by constitutive autocrine secretion of *gp130* family cytokines, and is functionally relevant because EpiSCs retain full competence to transduce these signals: while they express lower levels of the LIF-specific receptor *Lifr* relative to naive ESCs, they maintain robust expression of the shared *gp130* co-receptor required for all IL-6 family cytokine signaling. It will be intricate to identify the cytokines secreted by EpiSCs to maintain the basal activity of JAK/STAT3.

Importantly, in our previous study showed that addition of JAK inhibitor restrained LIF-induced primed-to-naive reprogramming. Critically, this effect was specific to the reprogramming process itself, as JAK inhibition had no impact on the self-renewal phenotype of already established naive Epi-iPSCs. SOCS3 is transcriptionally induced within 1 h of STAT3 activation in EpiSCs, which inhibits STAT3 phosphorylation levels before signal amplificated (Yang et al., 2010). In wild-type EpiSCs, this feedback maintains STAT3 activity at a level insufficient to overcome the second layer of regulation: chromatin-level restriction. As we have reported, the cBAF chromatin remodeling complex undergoes a functional switch during the naive-to-primed transition, dissociating from STAT3 and preferentially interacting with SMAD2/3 to drive primed gene expression (Ma et al., 2025). There exists a possible mechanism here. *Socs3* deletion may lead to the accumulation of p-STAT3 to supraphysiological level, which effectively competes with SMAD2/3 for the limited pool of cBAF complexes, shifting the transcriptional balance toward naive pluripotency.

Importantly, our findings are consistent with and extend our previous genome-wide characterization of STAT3 function in pluripotency. We have shown that STAT3 directly binds and represses the promoters of core primed genes (*Fgf5*, *Otx2*, *Dnmt3a*) to maintain naive identity, and that LIF withdrawal (STAT3 downregulation) leads to significant loss of the repressive histone mark H3K27me3 at these loci (Ma et al., 2025). The expression changes of these genes observed in *Socs3* KO EpiSCs in this study align with this STAT3 binding profile, providing independent confirmation that our phenotype is mediated by the canonical STAT3 pathway. While these data establish a solid mechanistic foundation, additional knockout EpiSCs lines for other SOCS family members and direct ChIP-seq analysis in *Socs3* KO EpiSCs will be needed to fully delineate the complete downstream transcriptional network and address dynamic temporal regulation during reprogramming.

This work has both limitations and translational potential. Restricted to mouse PSCs, it leaves the conservation of SOCS3’s barrier function in human pluripotent state transition unresolved. We have not performed *in vivo* chimera and teratoma assays to further validate the developmental potential of *Socs3*-deficient rnESCs, nor explored the functional role of SOCS3 in the formative pluripotent state that bridges naive and primed pluripotency (Carbognin et al., 2023; Smith, 2017).

In sum, our findings identify SOCS3 as a vital single target for optimizing stem cell fate control. SOCS3 also represents a promising target for small molecule inhibitors, with potential to enable reversible primed-to-naive pluripotency conversion and somatic reprogramming—critical for clinical stem cell applications.

## Data Availability

The original contributions presented in the study are publicly available. The Sanger sequencing data of the *Socs3* targeted genomic locus can be found at Figshare: https://doi.org/10.6084/m9.figshare.32756394. All other raw experimental data supporting the findings are available from the corresponding author upon reasonable request.
