# Supplementary material for: SOCS3 deficiency drives the primed to naive pluripotency transition by sustaining STAT3 activation
Source: Front Genet. 2026 Jul 20;17:1857225. doi: 10.3389/fgene.2026.1857225 (PMC13429239; doi:10.3389/fgene.2026.1857225)
Supplement: Supplementary file 1 [file Table1.docx]

**Supplementary Materials**

**Table S1. List of qRT-PCR primer sequences**

| Gene | Sequence | Sequence |
| --- | --- | --- |
|  | FW (5’-3’) | RV (5’-3’) |
| *Socs3* | GCGGGCACCTTTCTTATCC | AGGTGGCTGCTCCGGAACTT |
| *Nanog* | GCCTCCAGCAGATGCAAG | GGTTTTGAAACCAGGTCTTAACC |
| *Otx2* | GGTATGGACTTGCTGCATCC | CTCTCCCTTCGCTGTTTCC |
| *Fgf5* | AGAGTGGGCATCGGTTTCCATC | CCTACAATCCCCTGAGACACAG |
| *Gapdh* | CGTGCCGCCTGGAGAAAC | AGTGGGAGTTGCTGTTGAAGTC |
| *Rex1* | GAGACTGAGGAAGATGGCTTCC | CTGGCGAGAAAGGTTTTGCTCC |
| *Oct4* | AAGCTGCTGAAGCAGAAGAG | TTAAGGCTGAGCTGCAAGG |
| *Dnmt3b* | CCCTCCCCCATCCATAGT | TCTGCTGTCTCCCTTCATTGT |
| *Gata6* | GGTCTCTACAGCAAGATGAATGG | TGGCACAGGACAGTCCAA |
| *Gata4* | GGGCCCTCTTTGTCATTCTT | GGCTAAAGAAGCCTAGTCCTTG |
| *Prdm14* | GGCCATACCAGTGCGTGTA | TGCTGTCTGATGTGTGTTCG |
| *Tfcp2l1* | CAGCCTCTATCCAGGATGCACA | CTCTGGACATCTTCAGGAGGTC |
| *Pax6* | AGTGAATGGGCGGAGTTATG | GAACTGACACTCCAGGTGAAA |
| *Tuj1* | TAGACCCCAGCGGCAACTAT | GTTCCAGGTTCCAAGTCCACC |
| *Sox17* | TTGGCCGCGTCCATAAA | GACCTAACCCTTCGCCTAATC |
| *Brachyury* | CATTACACACCACTGACGCA | CATAGATGGGGGTGACACAG |

**Table S2. Antibody information**

| Antibody | Source | Cat# | Dilution |
| --- | --- | --- | --- |
| Mouse monoclonal Anti-Gapdh | Proteintech | Cat#60004-1-Ig | 1:10000 |
| Rabbit polyclonal Anti-Socs3 | Proteintech | Cat#14025-1-AP | 1:1000 |
| Mouse monoclonal Anti-Stat3 | Santa Cruz | Cat#sc-8019 | 1:1000 |
| Rabbit monoclonal Anti-p-STAT3(Tyr705) | Wanleibio | Cat#WL06214 | 1:1000 |
| Mouse monoclonal Anti-Gata4 | Santa Cruz | Cat#sc-25310 | 1:200 |
| Rabbit polyclonal Anti-Pax6 | Proteintech | Cat#12323-1-AP | 1:200 |
| Rabbit polyclonal Anti-alpha smooth muscle Actin | Abcam | Cat#ab5694 | 1:200 |

**Table S3. Reagent information**

| Reagent | Source | Cat# |
| --- | --- | --- |
| CHIR99021 | Tocris | Cat#4423 |
| PD0325901 | Tocris | Cat#4192 |
| LIF | Peprotech | Cat#300-05 |
| Activin A | Peprotech | Cat#120-14E |
| FGF2 | Peprotech | Cat#100-18B |
| Stattic | TargetMol | Cat#T6308 |
| Fibronectin | Millipore | Cat#FC010 |
| Gelatin | Sigma | Cat#G1890 |
| Accutase | Sigma | Cat#A6964 |
| β-mercaptoethanol | Sigma | Cat#444203 |
| Fetal bovine serum (FBS) | Gibco | Cat#30044333 |
| Penicillin–Streptomycin–Glutamine | Gibco | Cat#10378016 |
