# Supplementary material for: SOCS3 deficiency drives the primed to naive pluripotency transition by sustaining STAT3 activation
Source: Front Genet. 2026 Jul 20;17:1857225. doi: 10.3389/fgene.2026.1857225 (PMC13429239; doi:10.3389/fgene.2026.1857225)
Supplement: Supplementary file 2 [file DataSheet1.pdf]

Fig.1

GAPDH

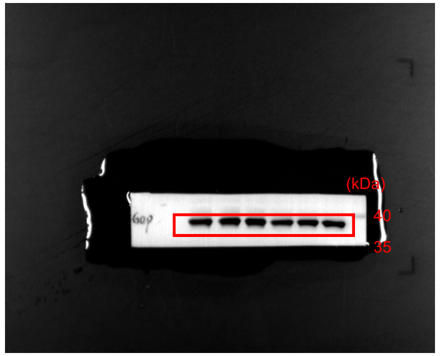

SOCS3

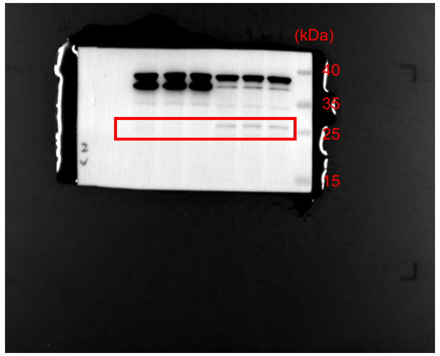

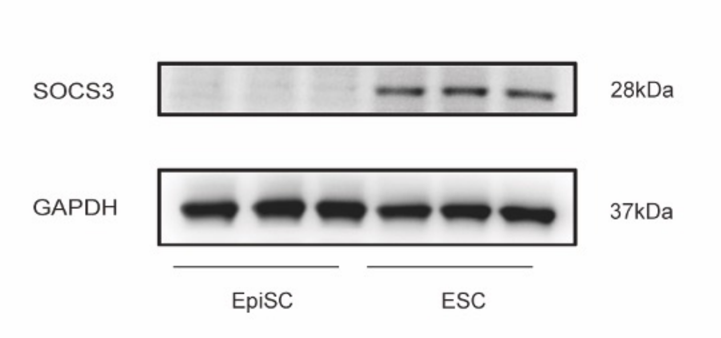

Fig.2

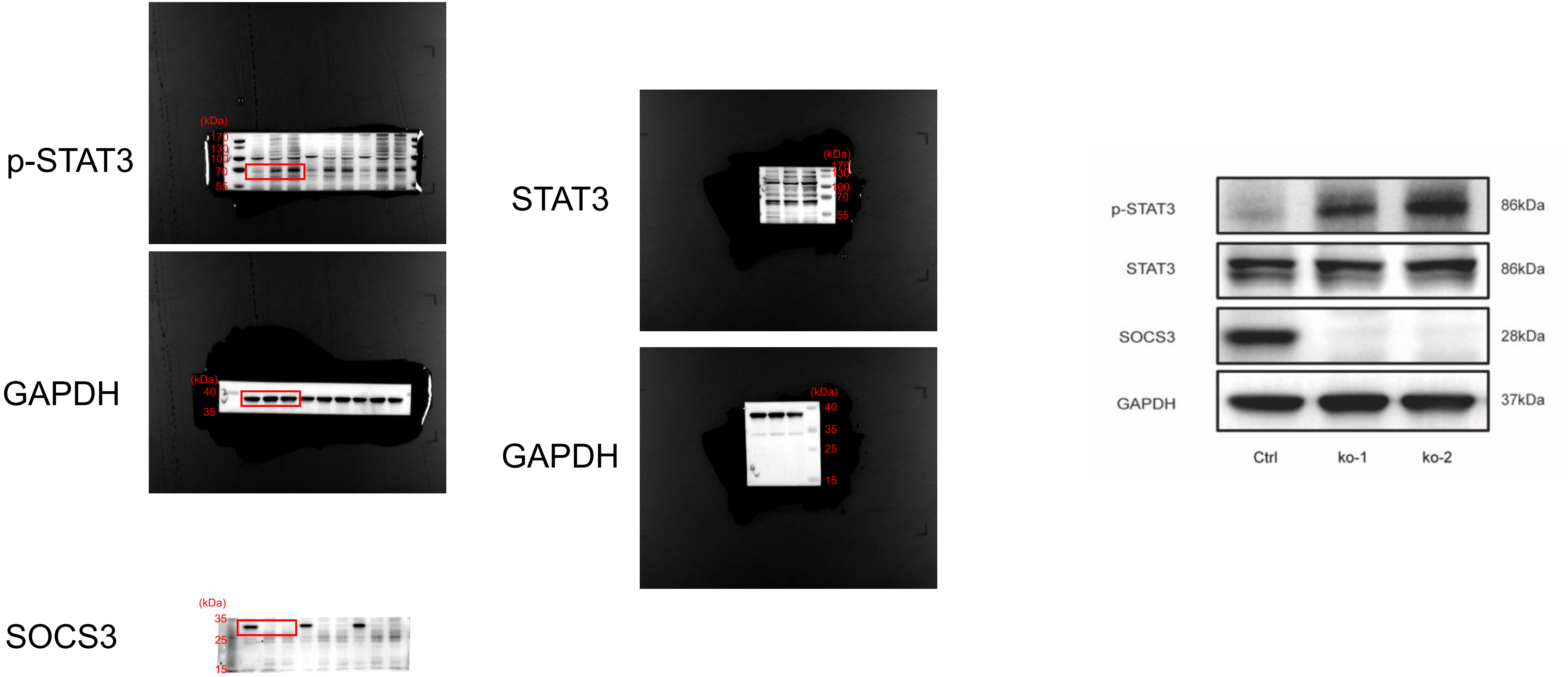
p-STAT3
GAPDH
SOCS3
STAT3
GAPDH
(kDa)
p-STAT3
86kDa
STAT3
86kDa
SOCS3
28kDa
GAPDH
37kDa
Ctrl
ko-1
ko-2

Fig.4

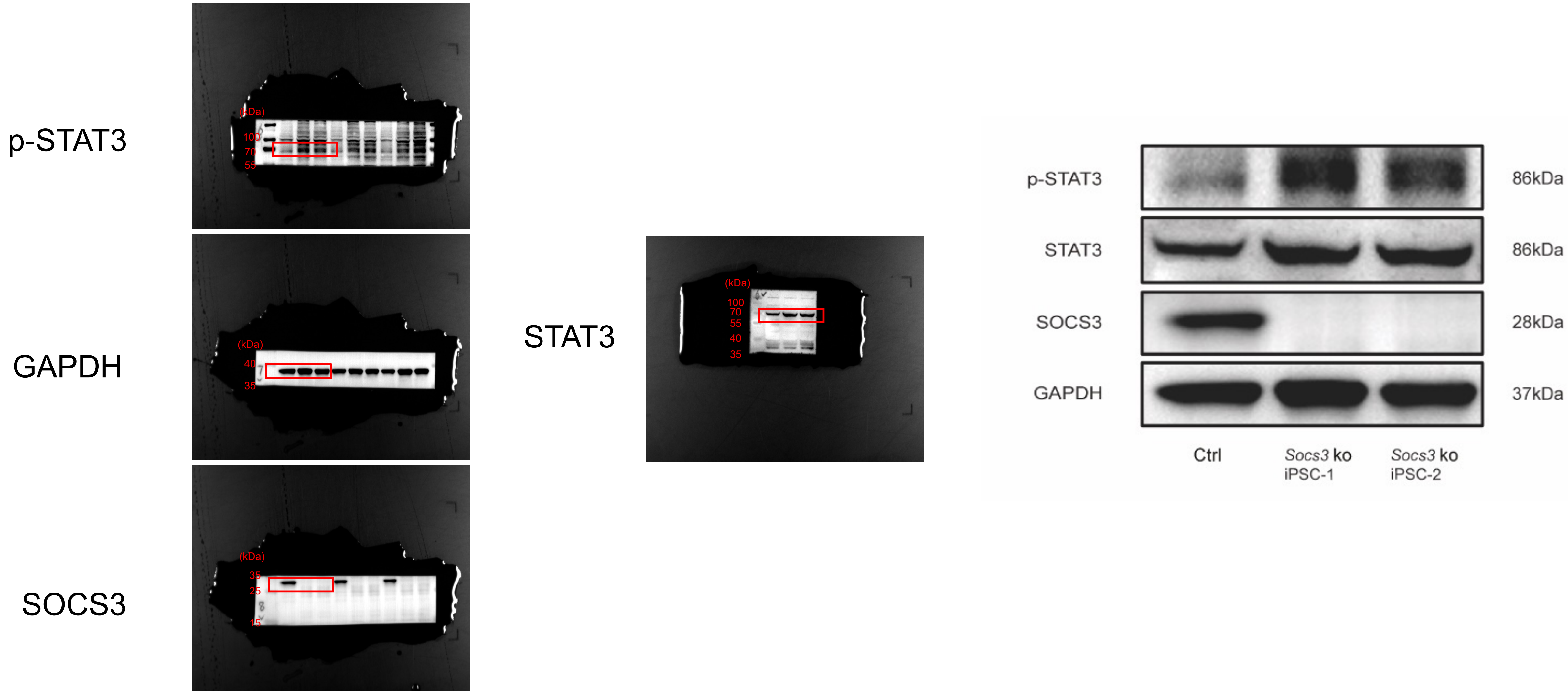
p-STAT3
(kDa)
100
70
55
GAPDH
(kDa)
40
35
SOCS3
(kDa)
35
25
15
STAT3
(kDa)
100
70
55
40
35
p-STAT3
86kDa
STAT3
86kDa
SOCS3
28kDa
GAPDH
37kDa
Ctrl
Socs3 ko iPSC-1
Socs3 ko iPSC-2

Fig.5

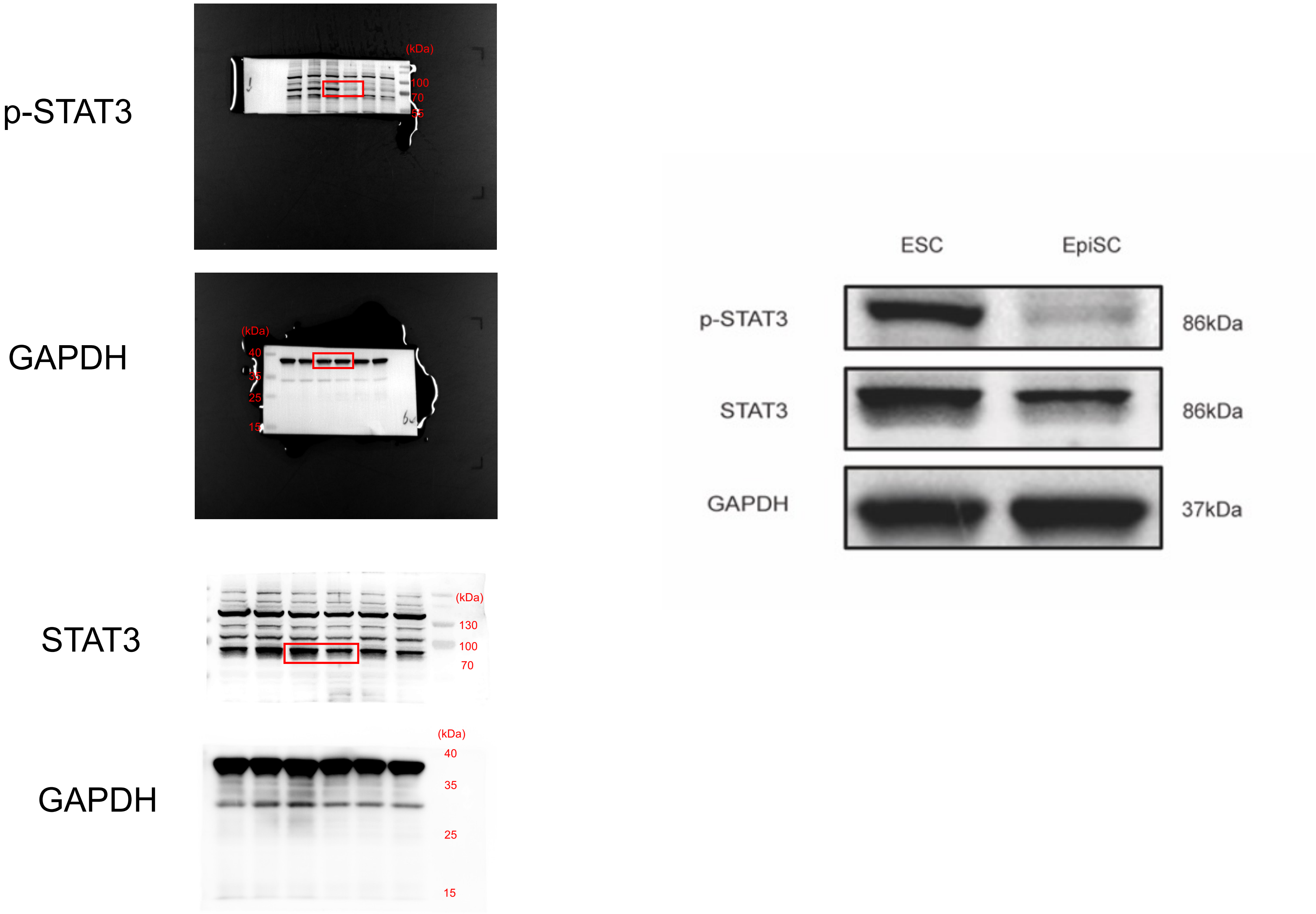
p-STAT3
GAPDH
STAT3
GAPDH
(kDa)
100
70
(kDa)
40
25
15
(kDa)
130
100
70
(kDa)
40
35
25
15
ESC
EpiSC
p-STAT3
STAT3
GAPDH
86kDa
86kDa
37kDa

Fig.5

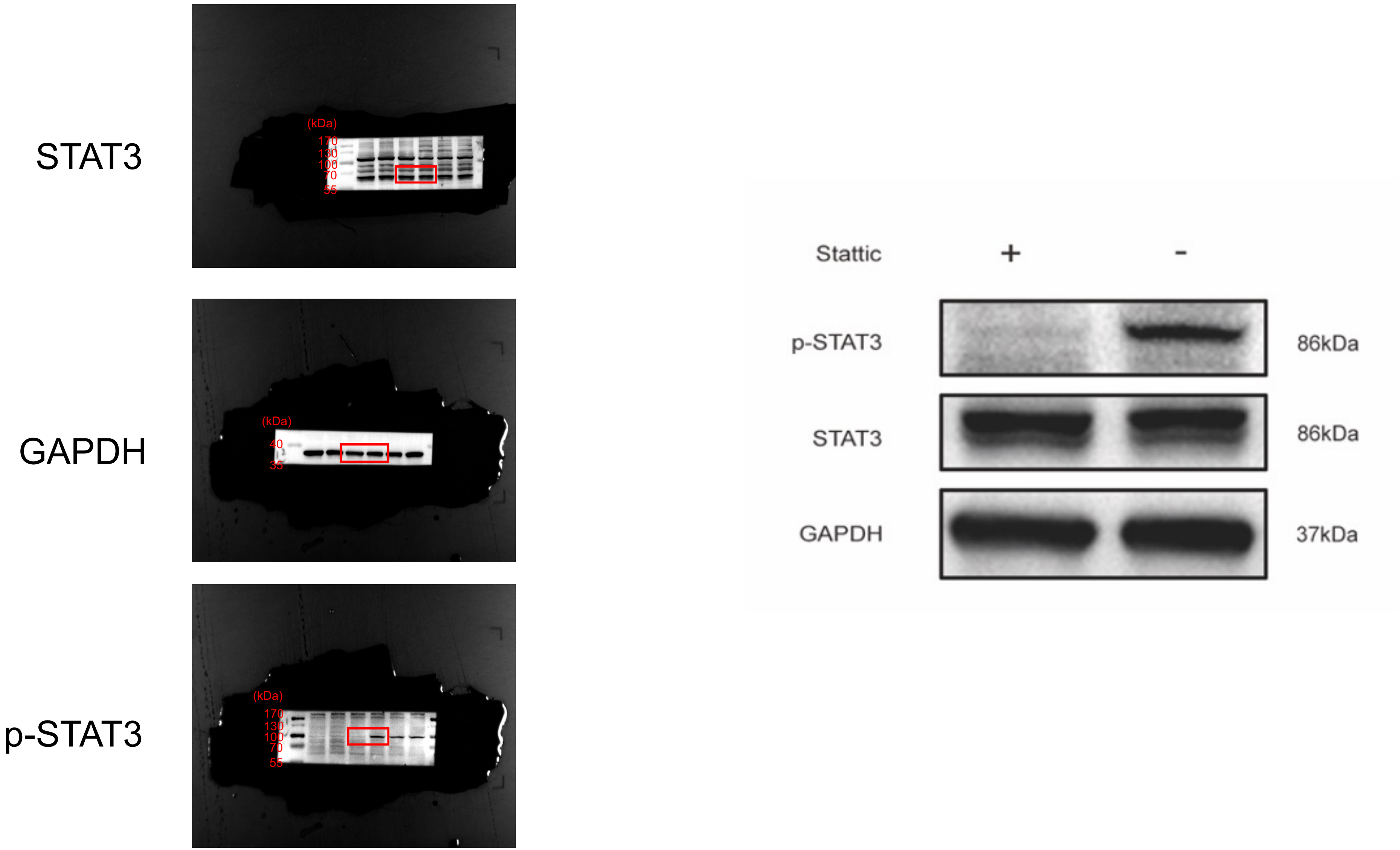
STAT3
(kDa)
170
130
100
70
55
GAPDH
(kDa)
40
35
p-STAT3
(kDa)
170
130
100
70
55
Stattic
+
-
p-STAT3
86kDa
STAT3
86kDa
GAPDH
37kDa
